# Responses of Intraspecific and Interspecific Trait Variations to Nitrogen Addition in a Tibetan Alpine Meadow

**DOI:** 10.3390/plants13131764

**Published:** 2024-06-26

**Authors:** Jialuo Yu, Peili Shi, Ning Zong, Minghua Song, Yujue Miao, Xiaofang Huang, Xueying Chen, Huixin Hei

**Affiliations:** 1 Institute of Geographic Sciences and Natural Resources Research, Chinese Academy of Sciences, Beijing 100101, China; 2 College of Resources and Environment, University of Chinese Academy of Sciences, Beijing 100190, China

**Keywords:** community weighted mean, community non-weighted mean, interspecific and intraspecific variation, nitrogen addition, alpine meadows

## Abstract

A community functional structure may respond to environmental changes such as nitrogen (N) enrichment by altering intraspecific and interspecific trait variations. However, the relative contributions of both components in determining the community response to N enrichment are unclear. In this study, we measured the plant height (H), leaf area (LA), leaf dry matter content (LDMC), and specific leaf area (SLA) based on a nine-year N addition gradient experiment in an alpine meadow on the Tibetan Plateau. We examined the intraspecific and interspecific variations within and among the communities, the responses of traits in terms of community weighted mean (CWM) and non-weighted mean (CM) to N addition, and the effects of these trait variations on aboveground net primary productivity (ANPP). Our results show that N addition increased the interspecific variation in H while decreasing that of LA within the community, whereas it had no significant effects on the intraspecific variations in the four traits within the community. In contrast, N addition significantly increased the intraspecific variation in H and decreased that of LA among the communities. Moreover, the contribution of intraspecific variation was greater than that of the interspecific variation in terms of CWM for all traits, while the opposite contribution was observed in terms of CM, suggesting that the dominant species would have greater resilience while subdominant species would become less resistant to N addition. Further, intraspecific variations of LA and LDMC within the community played an important role in explaining community productivity. Our results highlight the importance of both intraspecific and interspecific variations in mediating functional trait responses to N enrichment, and intraspecific variation within the communities has important implications for community functioning that should be considered to better understand and predict the responses of the alpine grasslands to N enrichment.

## 1. Introduction

Plant functional traits closely associated with plant growth and survival are essential for predicting community assembly and plant responses to environmental changes [1,2,3,4]. The responses of plant communities to environmental changes are generally quantified by a variety of metrics of functional trait composition [5,6]. Based on the biomass ratio hypothesis, community weighted mean (CWM) is calculated using the relative abundance of species in the community as weights [7]. The CWM highlights that the functional traits of dominant species are decisive for shaping community functions [8,9], and it has been widely used in studies of plant community responses to environmental changes [10,11,12]. In addition, community non-weighted mean (CM) is also commonly used to represent community-level values that are determined by the presence or absence of species rather than the relative abundance of species in the community [9,11,13]. The CM has contributed to highlighting the potential role of subdominant species in the community [5,13]. Therefore, comparing CWM and CM is beneficial for understanding the responses of dominant and subdominant species to environmental changes.

The phenotypic plasticity in plants allows it to have a flexible and rapid response to environmental changes [14,15]. As an important global change factor, N enrichment generally favors resource-acquiring species that have higher specific leaf area (SLA) and lower leaf dry matter content (LDMC) [16,17,18], which results in variations in plant traits. N enrichment regulates the functional trait composition of plant communities through intraspecific and interspecific variations [19,20,21]. Given that interspecific variation is thought to play a dominant role, most studies in terms of CWM and CM have focused primarily on interspecific variation, thereby neglecting the importance of intraspecific variation. However, a growing number of studies have shown that intraspecific variation plays an important role in the response of plant communities to environmental changes [22,23,24,25]. For instance, Jung et al. [26] found that intraspecific variation in SLA accounted for 44% of trait variation at the community level. A meta-analysis revealed that intraspecific variation was a decisive factor in determining plant responses to environmental changes, explaining 25% and 32% of the trait variation within and among communities, respectively [27]. Therefore, disentangling the relative contributions of intraspecific and interspecific trait variations is important to gain an understanding of how plant communities respond to environmental changes.

The intraspecific variations of plant traits play an important role in determining the functional composition of communities in response to N enrichment [14,28,29,30]. Li et al. [31] showed that nutrient addition increased the CWMs of plant height (H) and SLA in an alpine meadow, with these changes mainly driven by intraspecific variation under high nutrient addition. Meanwhile, N enrichment may enhance light limitation to the dwarf species [20,32], leading to a possible tendency for these shorter species to increase SLA in order to capture and utilize light for photosynthesis [28]. With increasing competition for light and nutrients, shorter species may gradually be eliminated, ultimately leading to the loss of community species [33,34,35]. In addition, field experiments in the typical grasslands of Inner Mongolia indicated that N enrichment significantly enhanced intraspecific trait variation and increased the width of niches between species, leading to reduced species richness [36].

Alpine meadows are the predominant grasslands on the Tibetan Plateau and are highly vulnerable and sensitive ecosystems to environmental changes [37,38]. To better understand the potential mechanisms by which species composition and productivity respond to N addition, a large number of N addition field experiments have been conducted in the alpine meadows on the Tibetan Plateau. However, it remains unclear how CWM and CM trait values affect aboveground net primary productivity (ANPP), as well as the relative contributions of intraspecific and interspecific trait variations across the N addition gradient. In this study, we quantified the contributions of intraspecific relative to interspecific variations in different functional traits along N addition gradient (see trait variation indices in Table 1). Four functional traits closely related to plant resource acquisition and community productivity were selected as follows: H, LA, LDMC and SLA. The main objectives of the study included the following: (1) determine the effects of N addition on intraspecific and interspecific variabilities in plant H, LA, LDMC, and SLA within the communities; (2) compare how the CWM and CM functional composition of the plant communities vary in response to N addition for different functional traits among the communities; and (3) quantify the relative contribution of intraspecific and interspecific variations to plant productivity following N addition.

## 2. Results

### 2.1. Trait Variations within Communities Induced by N Addition

**Figure 1:**
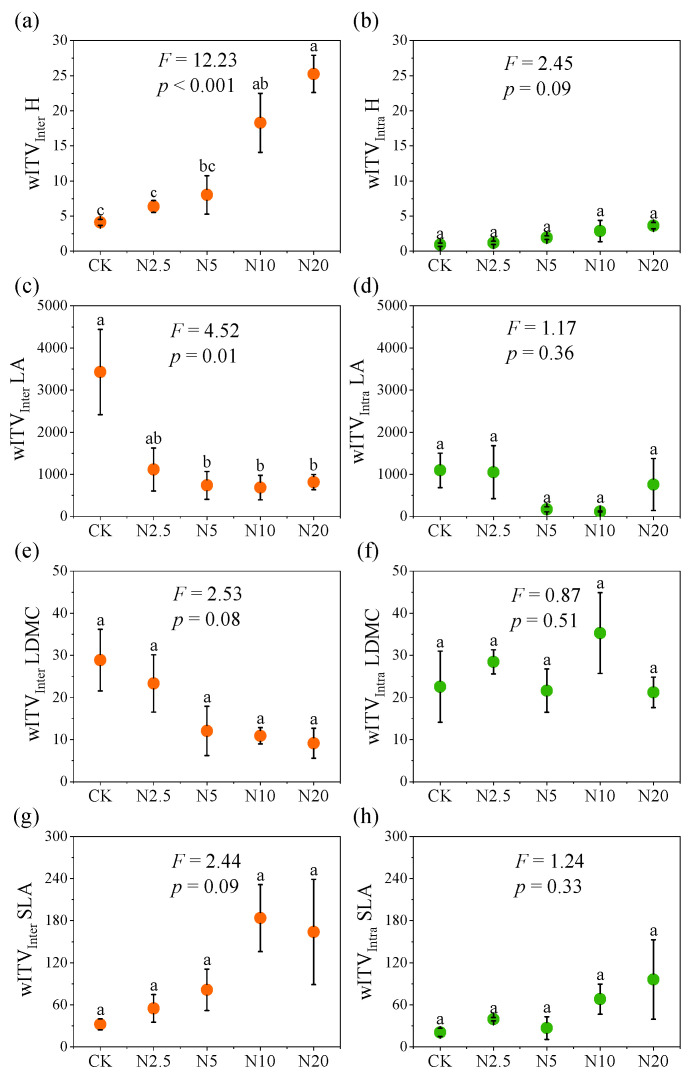
Interspecific (wITV_inter_) and intraspecific (wITV_intra_) variations in H (**a**,**b**), LA (**c**,**d**), LDMC (**e**,**f**),and SLA (**g**,**h**) within communities along the N addition gradient. H, plant height; LA, leaf area; LDMC, leaf dry matter content; SLA, specific leaf area. CK, N2.5, N5, N10, and N20 denote N addition of concentrations of 0, 2.5, 5, 10, and 20 g N m^−2^ year^−1^, respectively. Different letters indicate significant differences along the N addition gradient (Tukey’s test, *p* < 0.05). Vertical bars represent the standard error of means (n = 4).

Our results showed that N addition significantly altered interspecific trait variation within the community, while having no significant effect on intraspecific traits (Figure 1). Specifically, N addition significantly increased the wITV_inter_ of H (*p* < 0.001), which was significantly higher in N20 than in the treatments of CK and N2.5 (Figure 1a). In addition, N addition significantly decreased the wITV_inter_ of LA, as evidenced by the significantly lower values of wITV_inter_ of LA in the treatments of N5, N10, and N20 compared to the CK (Figure 1c). However, N addition had no significant effect on wITV_inter_ of LDMC and SLA (Figure 1e,g), as well as on the intraspecific trait variations for all four functional traits (Figure 1b,d,f,h).

### 2.2. Trait Variations within Communities Induced by N Addition

**Figure 2:**
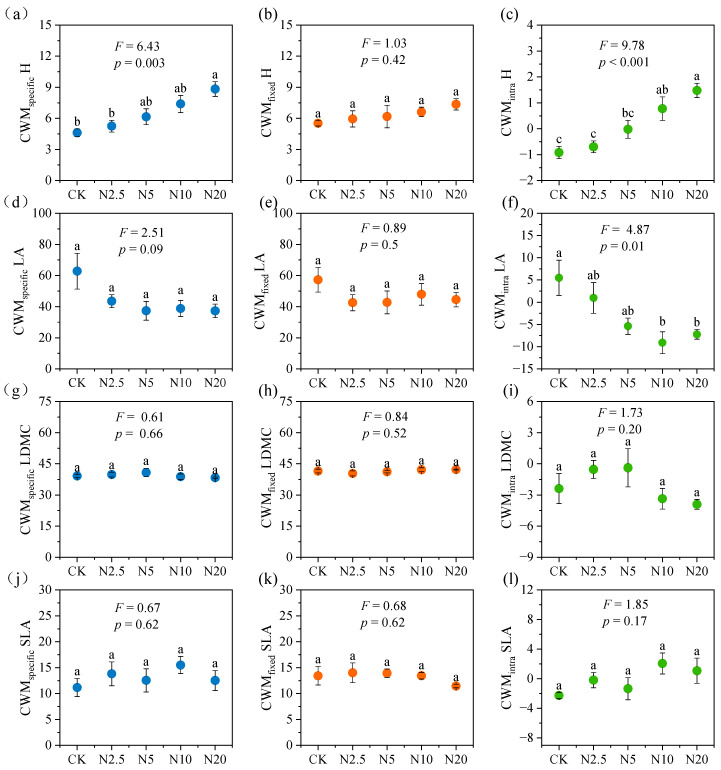
The specific, fixed, and intraspecific variations in H (**a**–**c**), LA (**d**–**f**), LDMC (**g**–**i**), and SLA (**j**–**l**) based on community weighted mean (CWM) among communities along N addition gradient. H, plant height; LA, leaf area; LDMC, leaf dry matter content; SLA, specific leaf area. CK, N2.5, N5, N10 and N20 denote N addition of concentrations of 0, 2.5, 5, 10, and 20 g N m^−2^ year^−1^, respectively. Different letters indicate significant differences along the N addition gradient (Tukey’s test, *p* < 0.05). Vertical bars represent the standard error of means (n = 4).

Based on the CWM, we calculated the variation in different functional traits among the communities along the N addition gradient (Figure 2). Our results showed that N addition significantly increased the CWM_specific_ of H, which was significantly higher in N20 than in CK and N2.5 (Figure 2a), whereas there were no significant effects on CWM_specific_ of LA, LDMC, and SLA (Figure 2d,g,j). In addition, our results showed no significant changes in the CWM_fixed_ of H, LA, LDMC, and SLA along the N addition gradient (Figure 2b,e,h,k). The CWM_intra_ of H significantly increased (Figure 2c), while CWM_intra_ of LA significantly decreased with increasing N addition (Figure 2f). N addition did not have a significant effect on CWM_intra_ for LDMC and SLA (Figure 2i,l).

**Figure 3:**
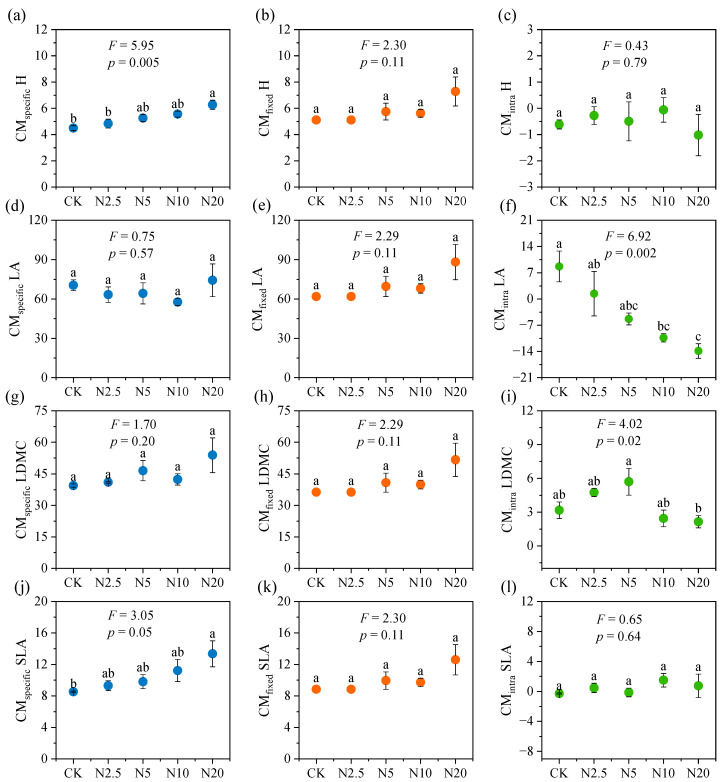
The specific, fixed, and intraspecific variations in H (**a**–**c**), LA (**d**–**f**), LDMC (**g**–**i**), and SLA (**j**–**l**) based on community non-weighted mean (CM) among communities along N addition gradient. H, plant height; LA, leaf area; LDMC, leaf dry matter content; SLA, specific leaf area. CK, N2.5, N5, N10 and N20 denote N addition of concentrations of 0, 2.5, 5, 10, and 20 g N m^−2^ year^−1^, respectively. Different letters indicate significant differences along the N addition gradient (Tukey’s test, *p* < 0.05). Vertical bars represent the standard error of means (n = 4).

Regarding the variations of different traits among the communities under N addition based on the CM, our results showed that N addition significantly increased the CM_specific_ of H and SLA (Figure 3a,j), while there were no significant effects on the CM_specific_ of LA and LDMC (Figure 3d,g). The CM_specific_ of H was significantly higher in N20 than in CK and N2.5, and the CM_specific_ of SLA was significantly higher than that of CK. In addition, our results showed no significant changes in CM_fixed_ for H, LA, LDMC, and SLA along the N addition gradient (Figure 3b,e,h,k). The CM_intra_ of H and SLA did not change significantly under N addition (Figure 3c,l), whereas the CM_intra_ of LA significantly decreased with increasing N addition (Figure 3f). The highest value of CM_intra_ of LDMC was found in N5 which was significantly higher than that of N20 (Figure 3i).

### 2.3. The Relative Importance of Intraspecific and Interspecific Trait Variation

**Figure 4:**
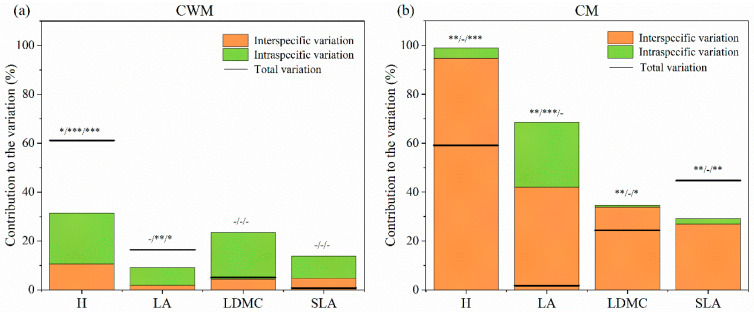
The decomposition of the total variability in plant functional traits (H, LA, LDMC, and SLA) explained by N addition into interspecific, intraspecific, and covariation effects for CWMs (**a**) and CMs (**b**) in alpine meadow. H, plant height; LA, leaf area; LDMC, leaf dry matter content; SLA, specific leaf area. Total variation is the sum of the interspecific, interspecific variation and covariation. Black bars represent total variation. The space between the black bars to the top of the column indicates the covariation effects. Positive covariance denotes that the bars are above the columns, and negative covariance denotes that the bars cross the columns. The asterisk at the top of the column indicates the significance value. The positioning of label (-, *p* > 0.05; *, *p* < 0.05; **, *p* < 0.01; ***, *p* < 0.001.) within the −/−/− graphic represents statistical significance of the interspecific/intraspecific/total variation, respectively.

By decomposing the total variance in terms of CWM, we found that the contributions of interspecific variations to trait variations were 10.5%, 1.8%, 4.5%, and 4.7%, and the contributions of intraspecific variations to trait variations were 20.9%, 7.3%, 19.0%, and 9.1% for H, LA, LDMC, and SLA, respectively (Figure 4a). There were positive covariances between the intraspecific and interspecific variation effects for H and LA, but negative covariances for LDMC and SLA. In contrast, when decomposing the total variance by CM, the contributions of interspecific variations to trait variation were 94.7%, 42%, 33.8%, and 26.9%, and the contributions of intraspecific trait variations were 4.2%, 26.6%, 0.8%, and 2.3% for H, LA, LDMC, and SLA, respectively (Figure 4b). In addition, there were negative covariations between the intraspecific and intraspecific variations effects for H, LA, and LDMC, but a positive covariance for SLA.

### 2.4. Explaining Intra- and Interspecific Trait Variation in ANPP

**Figure 5:**
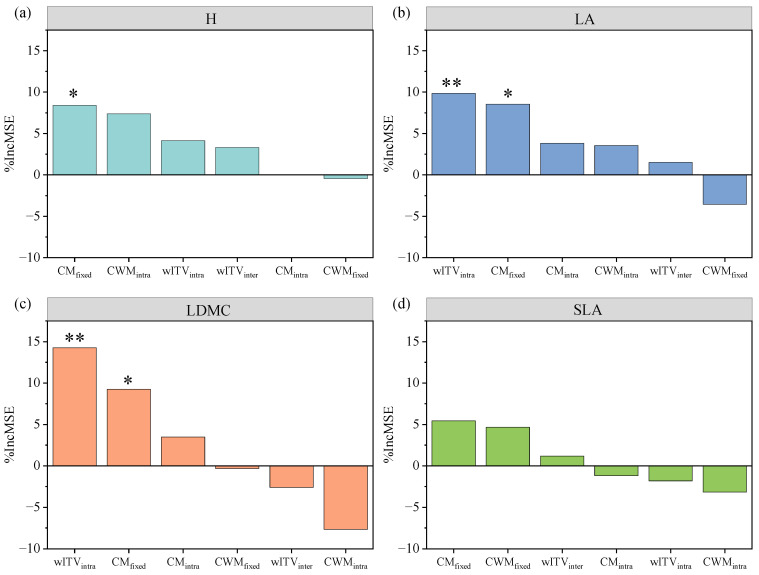
Random forest analysis predicts the relative importance of intraspecific and interspecific variations in explaining the variations in ANPP after N addition. H (**a**), plant height; LA (**b**), leaf area; LDMC (**c**), leaf dry matter content; SLA (**d**), specific leaf area. Percentage increases in mean squared error (% IncMSE) of variables were used to estimate the importance of these predictors, and higher % IncMSE values imply more important predictors. Asterisks denote the significant contribution to ANPP. *, *p* < 0.05; **, *p* < 0.01.

Our study showed that N addition had a significant effect on ANPP (*F* = 4.5; *p* = 0.013), which had the lowest ANPP at high nitrogen levels (N10 and N20) (Figure S1). For different functional traits, we used the random forest model to predict the influence of different trait variation variables on ANPP (Figure 5). The results show that CM_fixed_ of H was the most important variable to predict ANPP, explaining 8.4% (Figure 5a). wITV_intra_ and CM_fixed_ of LA were the most important variables predicting ANPP, explaining 9.9% and 8.5%, respectively (Figure 5b). For LDMC, wITV_Intra_ and CM_fixed_ were the most important variables for predicting N-induced changes of ANPP (Figure 5c). In addition, it was found that the trait variation of SLA had no significant effect on ANPP (Figure 5d).

## 3. Discussion

### 3.1. N-Induced Trait Variations within and among Communities

Intraspecific and interspecific trait variations can occur either within a single community or among different communities [33], and both have important impacts on ecosystem functioning [17,39], whereas the role of trait variation within a community is generally overlooked. Unlike trait variation among communities in which traits are measured for the same species growing in different communities, trait variation within a community is the difference in traits between individuals of the same species growing within the community [17,33]. In this study, N addition increased the interspecific variation of H but decreased that of LA within the community (Figure 1a,c). N addition promoted the rapid growth of N-responsive species within the community, and the increase in H of these species led to the slow growth of shorter species due to light limitation [33,40], which, in turn, led to an increase in interspecific variation in H within the community. In addition, some species were lost at high N levels (Figure S2), and the plant community was dominated by N-responsive species, which may led to a decrease in interspecific variation in LA within the community. For intraspecific variation within a community, the results showed that N addition had no significant effects on the intraspecific variations of the four functional traits (Figure 1b,d,f,h), which may be due to the similarity of the living conditions of different individuals within a specific community. Conversely, the results among the communities show that N addition significantly increased the intraspecific variation of H and decreased that of LA (Figure 2c,f). A possible explanation was that N additions caused grasses with small leaf areas to grow rapidly and then dominate the community due to the release from soil available N limitation and led to the loss of larger leafed species such as forbs.

Our results indicated different patterns among the four traits in terms of CWM in response to N addition (Figure 2). In this study, N addition increased the whole-plant traits such as H that were more susceptible to intraspecific variation than organ-level traits such as SLA and LDMC (Figure 2a,g,j). Previous studies have also confirmed that organ-level traits tend to be more conserved, whereas whole-plant traits are susceptible to environmental changes and show greater intraspecific variation [5,27,41].

### 3.2. Comparing CWM- and CM-Based Trait Variations in N-Induced

This study determined the response of the community traits in terms of CWM (Figure 2) and CM (Figure 3) to N addition in an alpine grassland ecosystem. Differences in the relative contributions of interspecific and intraspecific variation suggest that community dominant and subdominant species may differ in response to N addition [13]. Our results show that intraspecific variation contributed more than interspecific variation to the total community trait variation in terms of CWM for all four traits (Figure 4a), while the opposite was observed in terms of CM (Figure 4b). On the one hand, the plant species traits of the alpine meadow might have converged under the influence of the harsh alpine environment [19,42,43]. On the other hand, N addition would lead to an increasingly prominent role for dominant species in the community, i.e., selection effect. Both effects might result in a more important role in intraspecific variation in terms of CWM. However, the roles of subdominant species in the community were highlighted when CM was used [13], and the responses of these species to N addition might contribute substantially to the interspecific variation in the community.

The relative contributions of intraspecific and interspecific variations in plant traits reflect the community’s resistance to environmental change [13,44,45]. Previous studies have shown that when interspecific variation is lower than intraspecific variation, community resistance is stronger [13,46]. In this study, when CWM was used, the relative contribution of intraspecific variation was greater than that of interspecific variation (Figure 4a), indicating that dominant species played an important role in keeping community resilience. In contrast, interspecific variation in terms of CM was more important than intraspecific variation (Figure 4b), reflecting that the plant community became less resistant to N addition when the species composition was equally considered.

Exploring covariance between intraspecific and interspecific variations is crucial for understanding the responses of plant communities to environmental change [5,21,47]. Intraspecific and interspecific variations tending to change in the same direction, i.e., positive covariation, may render the community more responsive to environmental change, whereas intraspecific and interspecific variations changing in opposite directions, i.e., negative covariation, may cause less responsive to environmental change for the average trait of the community [5,21]. For example, there was positive covariation in H calculated in terms of CWM, indicating that N addition favored the growth of taller species as well as the growth of taller individuals in those species. Interestingly, we found opposite trends in covariations of H, LA, and SLA calculated in terms of CWM (Figure 4a) and CM (Figure 4b), which may be related to various species-specific responses to N addition.

### 3.3. Effects of Different Trait Variations on ANPP

Trait variation plays an important role in explaining changes in community productivity to environmental changes [31,48]. Previous studies have shown that an increase in H in terms of CWM contributes to productivity because communities dominated by taller species are generally more productive [33,49]. In this study, we found that CM_fixed_ of H had a significant effect on productivity (Figure 5a), which means that species diversity affects community productivity when equally considering species composition, i.e., in terms of CM. In addition, we found that intraspecific variation within the communities of LA and LDMC also played an important role in explaining community productivity (Figure 5b,c). SLA and LDMC are traits that are representative of strategies for acquisition, use and storage of resources [50,51,52]. Numerous studies have demonstrated that N addition may increase SLA and decrease LDMC [14,53], which are closely linked to community productivity. Therefore, our results suggest that intraspecific variation within communities has a crucial role in shaping community productivity to N addition and this effect is not ignorable.

## 4. Materials and Methods

### 4.1. Experimental Sites and Design

This study was conducted in an alpine meadow located in Nagchu County (31°34′ N, 92°34′ E) at an altitude of 4570 m on the Northern Tibetan Plateau. The experimental site was characterized by a sub-frigid semi-humid monsoon climate, with mean annual temperature of −0.29 °C and mean annual precipitation of 467.9 mm based on meteorological station data from 1981 to 2021. The soil is composed of sandy loam [54], and the dominant plant species are *Kobresia pygmaea*, *Stipa purpurea*, and *Carex moorcroftii*. The rate of N deposition in this area is approximately 1 g N m^−2^ year^−1^ [55,56]. To explore the process of grassland community response to N deposition, the experiment with five N addition concentrations of 0, 2.5, 5, 10, and 20 g N m^−2^ year^−1^ was conducted in 2013, representing control, 2.5, 5, 10, and 20 times of the background N deposition, respectively [56]. Each treatment had four 4 m × 4 m replicates using a completely randomized design and was fenced to avoid grazing. In this study, granular CO(NH_2_)_2_ was selected as N fertilizer due to its easily sourced and wide application in grassland restoration on the Tibetan Plateau. CO(NH_2_)_2_ was added in the early growing season (mid-July) each year.

### 4.2. Sample Collection and Determination

In August 2022, we recorded the number of species as well as the number of individuals and relative cover of each species in 1 m × 1 m quadrat randomly placed in the plots. In addition, the aboveground living materials of each species within a 0.5 m × 0.5 m quadrat were clipped at ground level and sorted by species into different envelopes. All plant samples were dried at 65 °C until constant weight, and they weighed up to 0.1 mg for species aboveground biomass estimation. ANPP is expressed as the sum of biomass of all species in each quadrat.

We used the relative biomass of species in the community as abundance and selected species with a cumulative relative abundance of over 90% in each quadrat with reference to the 2013–2021 community survey results to determine their H, LA, LDMC, and SLA. The selected species included *Kobresia pygmaea*, *Stipa purpurea*, *Carex moorcroftii*, *Stracheya tibetica* Benth., *Potentilla bifurca* Linn., and *Potentilla nivea* Linn. (Figure S3). A total of 20 quadrats (five N-added treatments × four replicates) were investigated. Specifically, approximately eight mature individuals of each species in each quadrat were selected to measure plant H, for a total of 852 individuals across 20 quadrats. Then, at least six healthy leaves per individual were selected to measure LA using a digital leaf area meter (LI-3000; LI-COR, Lincoln, NE, USA) within 24 h. The number of leaves selected was increased when the species had tiny leaves in order to minimize measurement error [37]. Leaf fresh weight was obtained from water-saturated leaves, and leaf dry weight was determined after drying at 65 °C until constant weight. Both the fresh and dry weight of the leaves were measured to 0.001 g. LDMC was calculated as leaf dry mass divided by leaf wet mass. SLA was calculated as LA divided by its dry mass (m^2^ kg^−1^). In addition, we collected soil samples from 0 to 15 cm to determine soil pH, total N (TN), NH_4_^+^-N, and NO_3_^−^-N content, and the results are shown in the Figure S4.

### 4.3. Intraspecific and Interspecific Trait Variations within Communities

Based on the relative biomass of each species within the community as weights, intraspecific (wITV_intra_) and interspecific (wITV_inter_) variations for each functional trait within a specific community were calculated as follows [17,21]:

(1) $$wITV_{intra}=\sum_{i=1}^{S}a_{i}*\frac{1}{Nind_{i}}\sum_{j=1}^{Nind_{i}}{\left(x_{ji}-x_{i}\right)}^{2}$$

(2) $$wITV_{inter}=\sum_{i=1}^{S}a_{i}*{\left(x_{i}-\sum_{i=1}^{S}a_{i}x_{i}\right)}^{2}$$

where *a_i_* denotes the relative biomass of species *i* within the community, *Nind_i_* represents the number of individuals of species *i* collected, *x_ji_* denotes the trait value of individual *j* of species *i*, and *x_i_* is the mean value of a trait of species *i* in the specific community. The smaller their values indicate a greater convergence of intraspecific or interspecific trait variations within the community.

### 4.4. Intraspecific and Interspecific Trait Variations among Communities

Community trait means were calculated as CWM and CM for all traits on a per plot basis. Intraspecific and interspecific variations in various functional traits among communities were calculated according to the method of Lepš et al. [21]. The method of the sum of squares (SS) decomposition was applied to disentangle the relative contributions of intraspecific and interspecific variations in the community level H, LA, SLA, in terms of both CWM and CM [21]. Based on analysis of variance, the total SS of both CWM_specific_ and CM_specific_ variation induced by N addition was decomposed into interspecific effect (SS_fixed_), intraspecific effect (SS_intra_), and covariation effect between them (SS_cov_), that is, SS_specific_ = SS_fixed_ + SS_intra_ + SS_cov_. The total trait variation among communities under different treatments was expressed as specific community weighted mean (CWM_specific_), which is mainly caused by interspecific variation (change in species composition, CWM_fixed_) and intraspecific trait variation (CWM_intra_), and were calculated as follows:

(3) $$\mathrm{CW}M_{\mathrm{specific}}=\sum_{i=1}^{S}a_{i}x_{i}$$

(4) $$\mathrm{CW}M_{\mathrm{fixed}}=\sum_{i=1}^{S}a_{i}x_{\mathrm{average}}$$

(5) $$\mathrm{CW}M_{\mathrm{intra}}=\mathrm{CW}M_{\mathrm{specific}}-\mathrm{CW}M_{\mathrm{fixed}}$$

where *a_i_* denotes the relative biomass of species *i* within the community, *S* is the number of sampled species in the community, *x_i_* represents the mean value of a trait for species *i* in the given community, and *x*_average_ the mean value of a trait for species *i* over all individuals in all communities. Unlike wITV_intra_, CWM_intra_ reflects the average trait variation of the same species across treatments rather than the trait variation of individuals. Larger CWM_intra_ values indicate greater variation in traits among communities.

In addition, when considering only the presence or absence of species in the community, i.e., each species had the same weight, the variation in intraspecific and interspecific variations for the CM was calculated as follows:

(6) $$CM_{\mathrm{specific}}=\frac{\sum_{i=1}^{s}x_{i}}{S}$$

(7) $$CM_{\mathrm{fixed}}=\sum_{i=1}^{S}x_{average}$$

(8) $$CM_{\mathrm{intra}}=CM_{\mathrm{specific}}-CM_{\mathrm{fixed}}$$

### 4.5. Data Analysis

One-way analysis of variance (ANOVA) with Tukey’s HSD test (*p* < 0.05) was used to determine the effects of N addition on different variables (i.e., wITV_intra_ and wITV_inter_ for each functional trait within a specific community; CWM_specific_, CWM_fixed_, CWM_intra_ CM_specific_, CM_fixed_ and CM_intra_ among community). All graphs were drawn using Origin 2023. Further, a random forest analysis of the main trait variance variables influencing ANPP was conducted using the “randomForest” package in R software. v.4.1.3, and the importance of the factors was estimated using the percentage increase in the MSE (mean square error) of the variables.

## 5. Conclusions

Our results indicated that different traits showed divergent patterns of trait variations in response to N addition. Specifically, N addition enhanced interspecific variability in the trait H but reduced that of LA within communities, without significantly altering intraspecific variations of four studies traits, while significantly increasing intraspecific variability of H and decreasing that of LA among communities. In addition, the contribution of intraspecific traits to total variation in traits was greater than interspecific variation when highlighting the roles of dominant species, i.e., in terms of CWM, whereas the contribution of interspecific variation was greater than intraspecific variation when equally considering species composition, i.e., in terms of CM. Further, intraspecific variation within the community of LA and LDMC played an important role in explaining community productivity. These results highlighted the importance of disentangling the role of intraspecific and interspecific variations in understanding the effects of N enrichment on plant community structure and ecosystem functioning.

## Figures and Tables

**Table 1:** Description of trait variation indices.

Abbreviations	Description
wITV_intra_	Intraspecific variability within a community
wITV_inter_	Interspecific variability within a community
CWM_specific_	Total trait variability based on community weighted trait mean among communities
CWM_fixed_	Interspecific variability based on community weighted trait mean among communities
CWM_intra_	Intraspecific variability based on community weighted trait mean among communities
CM_specific_	Total trait variability based on community non-weighted trait mean among communities
CM_fixed_	Interspecific variability based on community non-weighted trait mean among communities
CM_intra_	Intraspecific variability based on non-weighted trait mean among communities

## Data Availability

Data are available upon a reasonable request.

## Supplementary Materials

The following supporting information can be downloaded at: https://www.mdpi.com/article/10.3390/plants13131764/s1, Figure S1. Variations of aboveground net primary productivity (ANPP) along the N addition gradient. Figure S2. Variations of species richness along N addition gradient. Figure S3. Cumulative relative abundance based on species biomass along N addition gradient. Figure S4. Variations of pH, soil total N (TN) and NH_4_^+^-N and NO_3_^−^-N content along N addition gradient.
