# Supplementary material for: Responses of Intraspecific and Interspecific Trait Variations to Nitrogen Addition in a Tibetan Alpine Meadow
Source: Plants (Basel). 2024 Jun 26;13(13):1764. doi: 10.3390/plants13131764 (PMC11244433; doi:10.3390/plants13131764)
Supplement: Supplementary file 1 [file plants-13-01764-s001.zip › plants-3028469-supplementary.pdf]

Supporting information

Figure S1. Variations of aboveground net primary productivity (ANPP) along N addition gradient**.**

Figure S2. Variations of species richness along N addition gradient.

Figure S3. Cumulative relative abundance based on species biomass along N addition gradient**.**

Figure S4. Variations of pH, soil total N (TN) and NH_4^+-N and NO_3^--N content along N addition gradient**.**

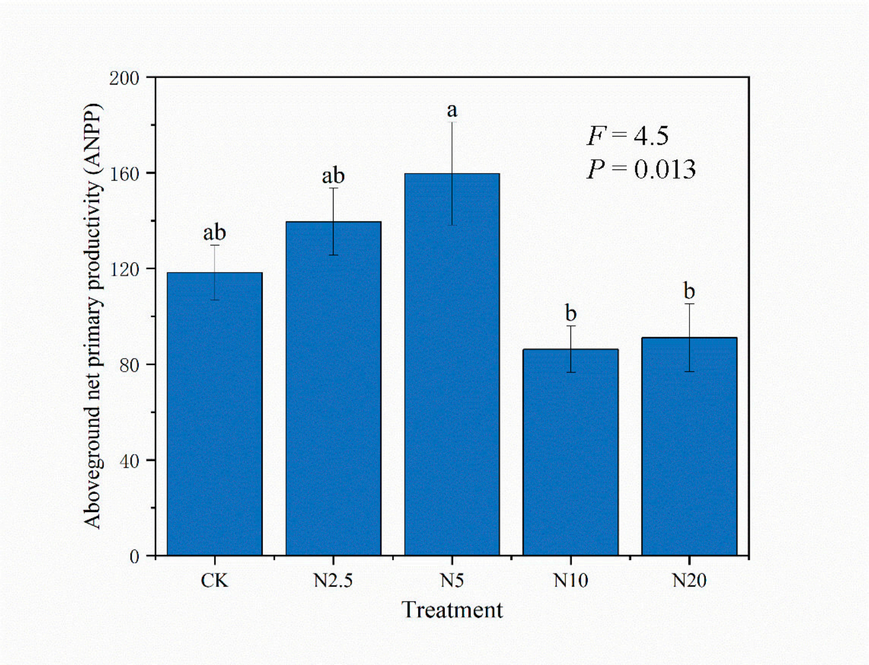

Figure S1. Variations of aboveground net primary productivity (ANPP) along N addition gradient. Different letters indicate significant differences among the four alpine grasslands (Tukey's test, $P < 0.05$).

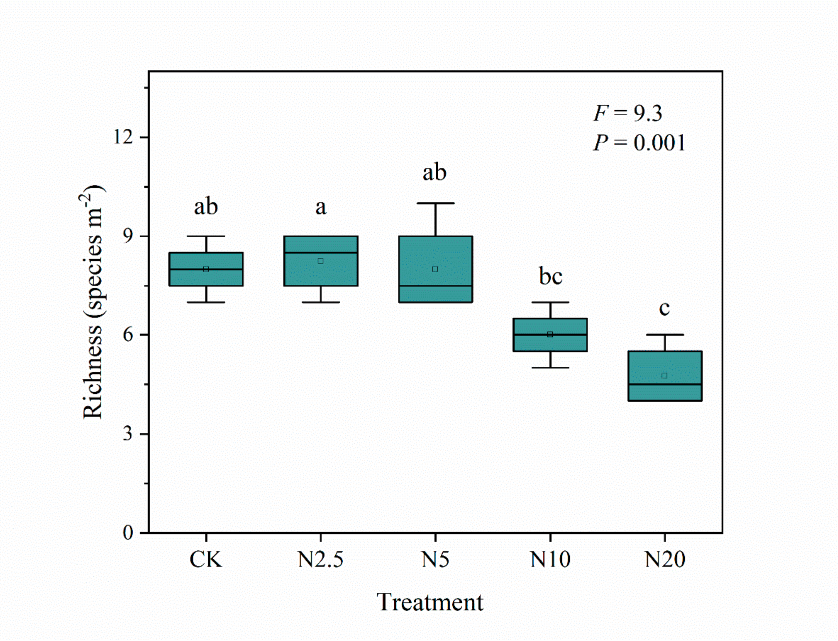

Figure S2. Variations of species richness along N addition gradient. Different letters indicate significant differences among the four alpine grasslands (Tukey's test, $P < 0.05$).

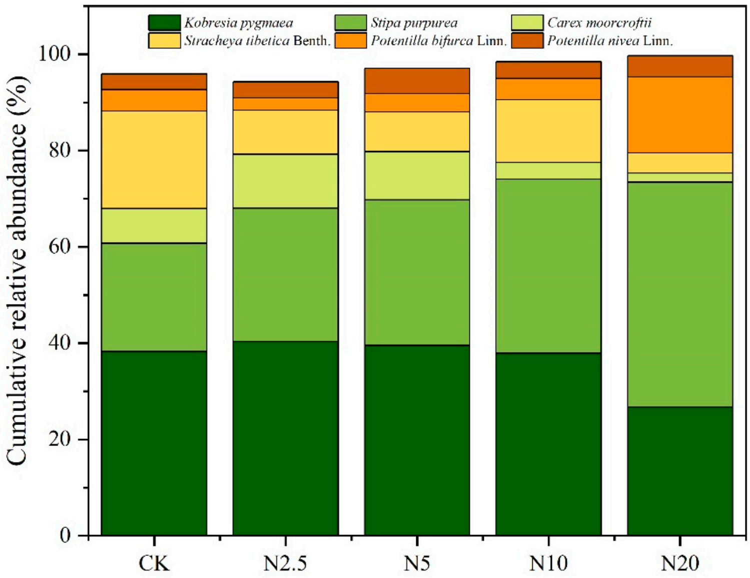

Figure S3. Cumulative relative abundance based on species biomass along N addition gradient**.**

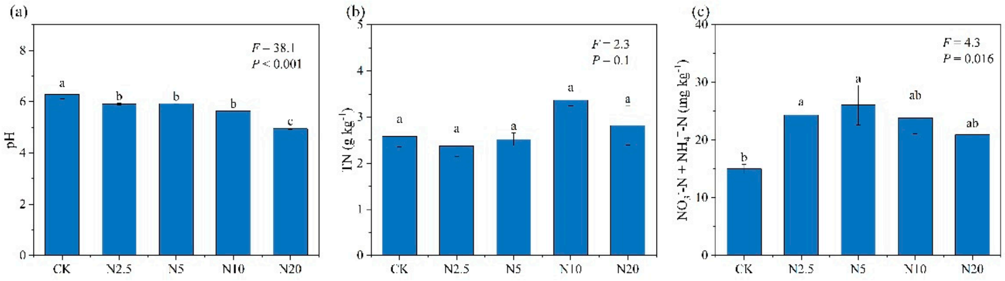

Figure S4. Variations of pH, soil total N (TN) and NH_4^+-N and NO_3^--N content along N addition gradient**.**
